# Lt.-General Burtchaell on American Medical Officers Serving with the British Armies in France

**Published:** 1918-09

**Authors:** 


### LIEUTENANT GENERAL BURTCHAELL ON AMERICAN MEDICAL OFFICERS SERVING WITH THE BRITISH ARMIES IN FRANCE.

The medical officers of the American Expeditionary Forces who were present at the recent meeting of the Research Society were thrilled with pride by the remarks of Lieut.-Gen. Burtchaell regarding the American physicians who are serving with the British armies in France. The fact as brought out by Gen. Burtchaell that our medical men have served with the same degree of gallantry, that they have suffered an equal proportion of casualties, and that they have been awarded military honors proportionate to those of the British medical officers with whom they are associated, is a splendid tribute to their courage and ability.

This expression of appreciation from Director General of the Medical Service of the British armies will be read with gratitude by the American Medical Officers serving under him, and with pleasure and interest by their confreres in the American Expeditionary Forces. The stenographic report of General Burtchaell's remarks is as follows:

" Perhaps I may be allowed to preface the discussion on War Wastage, which you have so kindly asked me to open, with a few words which do not directly bear on the subject. I refer to the medical units and the individual officers of the Medical Corps of the United States serving with the British armies in France. The complete personnel of two General Hospitals arrived for service with the British armies over two years ago, and during the last twelve months six Base Hospital units of the American Expeditionary Forces have been doing admirable work at various British bases. In addition some 700 individual officers of the United States Medical Corps have served as regimental medical officers in British units and in field ambulances and casualty clearing stations.

" I desire to take this opportunity of expressing my appreciation of the efficiency and good services rendered by these officers and by the Nursing Sisters and N. C. O.'s and Men of the Hospital units. They came to the British army and took up their duties amidst conditions and circumstances with which they were necessarily unfamiliar, but nevertheless they quickly adapted themselves to their work with a degree of facility, aptitude and efficiency which has won the admiration of everyone with whom they have come in contact. I have had the pleasure of meeting many of these officers myself. I should like to meet them all and thank each one personally.

" Those serving at the front have suffered losses, in common with others, and the ratio of battle casualties is practically identical with that of all medical officers of the British army in France during the war. Since your officers came to the British army twelve months ago, the percentage of losses among the officers of the R. A. M. C. was as follows;

| | R. A. M. C. | U. S. A. M. C. |
|---|---|---|
| Total losses. . . . . . . . . . . | 11.04 | 9.30 |
| In Front areas . . . . . . . . . | 12.66 | 12.74 |
| In Regimental Units. . . . . . | 12.35 | 12.35 |

" These very remarkable figures indicate that your officers were not wanting in carrying out their very arduous duties in the front line. From Commanding Officers and from all sections of the Medical Service I have frequently received reports of their excellent bearing and exemplary behavior.

" A number of officers have been specially mentioned in despatches; twenty-four have been awarded the Military Cross, and one officer has received a bar to his Cross. These awards have not been made because the recipients are members of the American Expeditionary Force and are consequently viewed from a different standpoint than that of British army officers. The reports on which these distinctions were awarded were rendered in exactly the same way as in the case of officers of the R. A. M. C., and the awards were made, irrespective of names, from the facts recorded describing some act of devotion to duty or gallant conduct, and by selection from a very large number of such reports.

" The number of honors gained by the officers of your Medical Corps indicates a very fine spirit and bearing. I give you these details, as many of you maynot other wise have heard of them. I desire once again not only to express my appreciation of the good service rendered by your officers, but also to express the hope that the good fellowship and association which exists between a considerable number of the officers of your Corps and the officers of the Royal Army Medical Corps may continue and develop into a still closer bond of sympathy and co-operation. "

